# Median Nerve Neural Mobilization Adds No Additional Benefit When Combined with Cervical Lateral Glide in the Treatment of Neck Pain: A Randomized Clinical Trial

**DOI:** 10.3390/jcm10215178

**Published:** 2021-11-05

**Authors:** Daniel Martin-Vera, Josué Fernández-Carnero, David Rodríguez-Sanz, Cesar Calvo-Lobo, Ibai López-de-Uralde-Villanueva, Alberto Arribas-Romano, Pedro Martínez-Lozano, Daniel Pecos-Martín

**Affiliations:** 1 Faculty of Sport Sciences, Universidad Europea de Madrid, Villaviciosa de Odón, 28005 Madrid, Spain; daniel.martinvera@gmail.com (D.M.-V.); pedro.martinez@universidadeuropea.es (P.M.-L.); 2 Department of Physical Therapy, Occupational Therapy, Rehabilitation and Physical Medicine, Rey Juan Carlos University, 28922 Madrid, Spain; alberto.arribas@urjc.es; 3 La Paz Hospital Institute for Health Research, IdiPAZ, 261, 28046 Madrid, Spain; 4 Grupo Multidisciplinar de Investigación y Tratamiento del Dolor, Grupo de Excelencia Investigadora, URJC-Banco de Santander, 28922 Madrid, Spain; 5 Motion in Brains Research Group, Institute of Neuroscience and Movement Sciences (INCIMOV), Centro Superior de Estudios Universitarios La Salle, Universidad Autonóma de Madrid, 28049 Madrid, Spain; 6 Grupo de Investigación de Dolor musculoesqueletico y Control Motor, Universidad Europea de Madrid, 28005 Madrid, Spain; 7 School of Nursing, Physiotherapy and Podiatry, Universidad Complutense de Madrid, 28606 Madrid, Spain; davidrodriguezsanz@ucm.es (D.R.-S.); cescalvo@ucm.es (C.C.-L.); 8 Department of Radiology, Rehabilitation and Physiotherapy, Universidad Complutense de Madrid, 28606 Madrid, Spain; ibailope@ucm.es; 9 Escuela Internacional de Doctorado, Department of Physical Therapy, Occupational Therapy, Rehabilitation and Physical Medicine, Universidad Rey Juan Carlos, 28933 Alcorcón, Spain; 10 Physiotherapy and Pain Research Center, General Foundation of the University of Alcalá, 28805 Madrid, Spain; daniel.pecos@uah.es; 11 Department of Physical Therapy, Alcalá University, 28805 Alcalá de Henares, Spain

**Keywords:** brachial plexus neuritis, chronic neck pain, musculoskeletal manipulations, rehabilitation, upper extremity

## Abstract

Background: This study aimed to compare the effectiveness of cervical lateral glide (CLG) added to median nerve neural mobilization (MNNM) in patients with neck pain (NP). Methods: A single-blinded randomized controlled clinical trial was carried out in a Pain Management Unit from a Hospital. A total sample of 72 patients with NP was recruited from a hospital. Patients were randomized to receive isolated CLG (*n* = 36) or CLG + MNNM (*n* = 36). Bilateral elbow extension range of motion (ROM) on upper limb neurodynamic test 1 (ULNT1), bilateral pressure pain thresholds (PPT) on the median nerve at elbow joint, C_6_ zygapophyseal joint and tibialis anterior, Visual analogue scale (VAS), body chart distribution of pain, active cervical ROM (CROM), Neck Disability Index (NDI), and Tampa Scale of Kinesiophobia (TSK-11) were measured at baseline as well as immediately, 15 days, and 1 month after treatment. Results: There were no statistically significant interactions (*p* > 0.05) between treatment and time for median nerve mechanosensitivity outcomes, pain intensity, symptom distribution, and PPT of the widespread pain assessment, as well as cervical function, and kinesiophobia. Conclusions: MNNM gave no additional benefit to CLG in patients with NP regarding pain intensity, symptom distribution, mechanosensitivity, functionality, and kinesiophobia. Only two treatment sessions and the short follow-up are important issues, therefore, justifying further studies to answer the research question with better methodology.

## 1. Introduction

Neck pain (NP) is a common disabling condition that is likely to affect most people at some time in their lives [1,2,3]. NP might negatively impact the social, familial, and work environments of the patients, as well as affect the health care system and contribute to economic burden [1,4,5,6]. The NP global point prevalence was 4.9% and the disability-adjusted life years showed an increase from 23.9 million in 1990 to 33.6 million in 2010. NP is ranked fourth among years lived with disability and 21st when considering the overall burden [3]. Indeed, the prevalence of NP has risen to 8.56% in Spain and reached 10.61% in conjunction with low back pain [1].

Neuropathic features might be present in 6.9% to 10% of patients with NP [7]. Indeed, neuropathic pain frequency might increase by up to 19.9% in patients with NP associated with pain in the arm (cervicobrachial pain (CBP)) [8]. Pain management among patients with nociceptive or neuropathic NP can be complex. Thus, the appropriate interventions to treat these patients depend on the nature of pain [9]. Although different interventions have been proposed for nociceptive [10,11] and neuropathic [12,13,14] NP, there is low evidence to determine the effectiveness of physical medicine modalities, such as neural mobilization, and high quality randomized clinical trials are necessary for patients with NP [15,16].

Regarding neural mobilization approaches for musculoskeletal conditions in patients with CBP [15,16], median nerve neural mobilization (MNNM) and cervical lateral glide (CLG) were shown to be more effective in short-term for improving pain intensity, range of motion (ROM) during upper limb neurodynamic test (ULNT1), and functionality than therapeutic ultrasound [17], manual cervical traction [18] or no treatment [19,20]. Although pharmacologic treatment, such as oral ibuprofen, can decrease pain intensity and disability more than MNNM or CLG, the non-existence of ROM differences between these interventions, as well as adverse effects associated with the pharmacological agents, suggest neural mobilization as a possible treatment option in patients with CBP [21,22]. Indeed, neural mobilization techniques for upper quadrant pain syndromes might modulate central sensitization and mechanosensitivity in patients with NP [23].

To our knowledge, the effectiveness of combining MNNM with CLG has not yet been determined in patients with NP. This study aimed to compare the effectiveness of CLG plus MNNM (vs. MNNM alone) on median nerve mechanosensitivity, intensity and distribution of NP, local/remote pressure pain threshold (PPT), cervical function, and kinesiophobia in patients with NP.

## 2. Materials and Methods

### 2.1. Trial Design

The study protocol was conducted from April to June of 2017 as an interventional phase II, parallel, single-blind, randomized controlled clinical trial. The study was conducted at the Pain Management Unit from the Alcala de Henares Hospital (Spain) in accordance with the CONSORT statement [24].

All of the procedures used in this trial were programmed according to the ethical principles of the Declaration of Helsinki [25]. All subjects were informed and provided informed consent to participate. This trial was approved by the Ethics Committee for Clinical Research of the Principe de Asturias Hospital (Code: OE 19/2013). This trial was prospectively registered in the Australian New Zealand Clinical Trials Registry (ACTRN12c617000430336).

### 2.2. Randomization and Blinding

The randomization and allocation of the patients to the trial group were done using computer software (Epidat^®^ 4.2 version, Xunta de Galicia, Spain) and randomized printed cards contained in consecutively numbered opaque sealed envelopes kept in a locked drawer unit. A member of the research team who was not involved in the assessment and/or treatment procedures performed the randomization and maintenance of the list. Treatment and outcome assessments were performed in different rooms and at different time points.

### 2.3. Follow-Up

The outcomes assessment was carried out at baseline, post-intervention, and 15 days and 30 days after the first intervention. Median nerve mechanosensitivity, pain-related outcomes, and cervical ROM were recorded in all evaluation time spots. Neck Disability Index (NDI) and the Tampa Scale of Kinesiophobia (TSK-11) measurements were not obtained at post-intervention. Sociodemographic data were also collected.

### 2.4. Median Nerve Mechanosensitivity

ROM in elbow extension on the ULNT1 measures was performed [26]. In the ULNT1 maneuver for fixing the shoulder girdle, wrist, and hand [26,27,28,29], patients needed to specify the moment in which sub-maximum pain level was reached with elbow extension, which means the level of pain that subjects can tolerate. Elbow extension was assessed using a universal goniometer (Sammons Preston^®^, Bolingbrook, IL, USA). UNLT1 Intra and inter-observer reliability is considered from good to excellent (ICC ≥ 0.98) [30]. An average of three measurements was calculated.

### 2.5. Widespread Pain Assessment

#### 2.5.1. Pain Intensity

Pain intensity levels were recorded with a VAS, described as a 100 mm non-segmented line, oriented horizontally, with both extremes labeled as “no pain” at its start and finishing with “the worst pain unimaginable”. Subjects were asked to rate their current pain with a mark on the scale [31]. VAS is a reliable (ICC) intraclass correlation coefficient and valid tool to measure pain levels. Test-retest reliability is high (ICC 0.71–0.99) [32,33].

#### 2.5.2. Body Pain Distribution

Pain/Symptoms distribution data was gathered using four body charts depicting the neck and upper limbs and all drawings were converted to a digital format. The percentage of symptoms change over time was analyzed using Matlab^®^ R2016a version (The MathWorks Inc, Natick, MA, USA) [34]. ICC for distribution of symptoms over the neck region is very high (ICC = 0.92), and the software error measured between two exact figures was 5.4%, with good test-retest reliability [34].

#### 2.5.3. Pressure Pain threshold (PPT)

PPT was used to quantitatively evaluate local and central tissue mechanosensitivity [33,35,36,37,38]. The PPT is defined as the lowest pressure that needs to be applied to cause the slightest sensation of pain [39]. Recordings were collected bilaterally over the articular pillar of the C6 zygapophyseal joint, median nerve at the cubital fossa of the elbow, and tibialis anterior muscle belly (5 cm distal from anterior tibial tuberosity and 2.5 cm lateral) [40,41,42,43]. A pressure algometer [FORCE DIAL FDK/FDN 100 model, Wagner Instruments (P.O.B. 1217, Greenwich, CT, USA)] was used (kg/cm^2^). ICC for the test-retest reliability of PPT in patients with NP are high (ICC: 0.83 to 0.89) [44,45]. In addition, the intra-examiner reliability (ICC = 0.94–0.97) is excellent [46]. An average of three measurements was calculated [47].

### 2.6. Cervical Function

Active cervical ROM was assessed with a CROM device (Cervical Range of Motion Instrument, Performance Attainment Associates, Roseville, MN, USA) through a standardized protocol. Patients were seated on a chair with the CROM device placed over the head. The assessor asked patients to perform active neck movements in the maximum range. ICC values are high for both intra-observer (ICC 0.92–0.96) and inter-observer (ICC 0.82–0.94) reliability. Among NP patients, SE has been reported as between 2.5° and 4.1° [48]. Three measurements were performed in each direction, and the average values were calculated.

Neck disability was measured with the NDI, which is a well-validated 10-item questionnaire, with each item rated on a 0 to 5 point scale. The highest results correspond to elevated levels of disability [49], NDI is the most used and validated instrument to measure disability levels in non-specific NP patients with or without irradiation symptoms [50,51]. A Spanish version of the NDI was utilized in this study [52].

### 2.7. TSK-11

TSK-11 was selected in this trial to quantify fear of movement behavior or vulnerability related to NP [53]. A validated and reliable Spanish version of the TSK-11 was used [54]. This version has demonstrated good psychometric properties [55].

### 2.8. Participants

Patients with NP were included in the study if they met the following inclusion criteria: between 18–65 years old; NP from non-traumatic origin with a clinical evolution of a minimum of 4 weeks [56] located from the superior nuchal line to the drawn segment between the superomedial angles of the scapula, with or without radicular symptoms radiated to the head, trunk, and/or the upper limbs [57]; and needed to understand, write, and speak Spanish fluently. Exclusion criteria following a clinical evaluation were: two or more abnormal neurological findings within C5-T1 levels; and central or peripheral neurological syndromes; pregnancy and patients that have received physiotherapy at the spine or glenohumeral region over the last 6 weeks.

### 2.9. Intervention

Each subject came to the center for 3 days. Four evaluation points and two interventions were carried out. These interventions were performed at the beginning of the trial and 2 weeks after.

Subjects were randomly and equitably allocated into two groups. A physiotherapist with more than 8 years of experience in manual therapy and neurodynamic techniques. Both groups received treatment of four series of Maitland grade 3 mobilizations [58] in 1 min with 1 min of rest between sets at a frequency of 0.5 Hz (with metronome control/steps). Both groups were taught to do the same neurodynamic exercises of median nerve sliding with movements of the head and elbow simultaneously as a home treatment program between sessions. Assurance of performance of home exercises was supported by an explanatory video sent by email and reminder calls from the trial staff to clarify doubts and motivate about the importance of completing the program [59]. The home exercises pattern was three sets of 1 min with 1 min of rest every day.

### 2.10. Cervical Lateral Glide and Median Nerve Mobilization (CLG + MNNM)

This group received a treatment of cervical lateral glide contralateral to the side where each subject suffered higher pain intensity. Patients lay in a supine position on a stretcher and ULNT1 was performed on the upper limb until subjects felt tension on the anterior part of the forearm or hand. Shoulder depression was controlled by a wooden device covered with a soft cushioning, preventing the elevation of the glenohumeral joint, as well as a wrist and thermoplastic splint held hand extension. The physiotherapist performed a contralateral cervical glide with both hands while the patient synchronously slightly flexed the elbow joint. When the therapist returned to the midline, the patients extended the elbow back to the starting position.

### 2.11. Cervical Lateral Glide in Isolation (Isolated CLG)

Like the previous group, this group received an intervention of cervical lateral glide but with either upper limb in the resting position, placing on the abdominal region.

### 2.12. Sample Size

The sample size was calculated using G*Power 3.0.18 Software (Bonn, Germany). A repeated-measures analysis of variance (ANOVA) was used to detect differences in the median nerve mechanosensitivity. A significance level of 5% α error and a power of 90% (1-β error) were also used. Moreover, a moderate effect size (*f* = 0.2) and sphericity correction of 0.75 were considered based on a previous pilot study. Assuming a 20% dropout rate, it was estimated that 68 participants were required (34 per group).

### 2.13. Statistical Analysis

Descriptive statistics were used to describe the baseline characteristics of each group. No statistically significant differences between groups were found. Were examined for normality using the Kolmogorov–Smirnov test for all variables, confirming that all variables were normally distributed. However, two-way ANCOVA was performed to control for the age of the participants because this variable showed differences between groups at baseline to median nerve mechanosensitivity, PPT, ROM, and self-administration questionnaires. The between-subject factor was the treatment (CLG + MNM treatment, CLG treatment), with time (baseline, 2 min immediately post-treatment, 2 weeks follow-up, 4 weeks follow-up) as within-subject factors. The effect size was calculated as the Partial Eta Squared (η^2^_p_) when significant. An effect size of 0.01 was considered small, 0.06 medium, and 0.14 large. A multiple comparisons analysis with Bonferroni adjustment was performed. Statistical analyses were performed using SPSS 22 (SPSS Inc, Chicago, IL, USA). The significance level was set at *p* < 0.05.

## 3. Results

**Figure 1:**
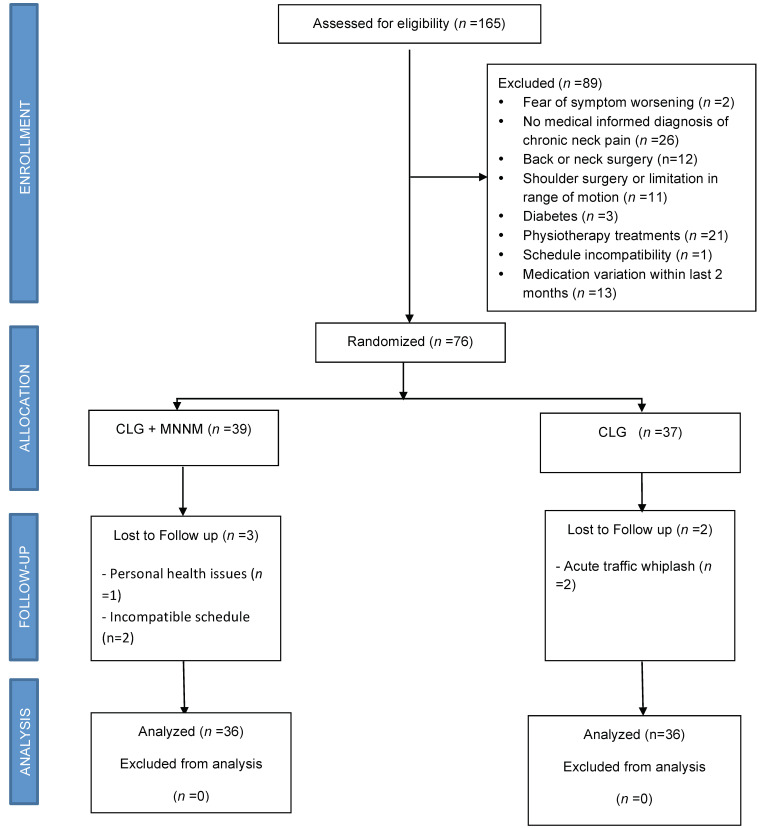
Consort flow diagram. CLG = cervical Lateral Glide; MNNM = median nerve neural mobilization.

The patients and the final inclusion are represented in the flow diagram (Figure 1). We initially recruited 165 patients, but finally, a total of 72 patients were definitively analyzed (29 males/43 females). Finally, 36 subjects completed the study in the cervical lateral glide (CLG) group and 36 CLG + median nerve mobilization (CLG + MNM) group. All of these participants completed the two treatment sessions, including the home task performance of neural mobilizations. The baseline characteristics of both groups are presented in Table 1. There were no significant differences in baseline variables between the groups (*p* > 0.05), except age (Table 1).

### 3.1. Median Nerve Mechanosensitivity

By two-way ANCOVA, we detected statistically significant differences only in the group factor for the contralateral median nerve PPTs (F = 5.767; *p* = 0.019; η^2^_p_ = 0.078). There were no statistically significant interactions between treatment and time for median nerve mechanosensitivity outcomes [homolateral ULNT1 (F = 1.118; *p* = 0.332; η^2^_p_ = 0.016); contralateral ULNT1 (F = 0.346; *p* = 0.747; η^2^_p_ = 0.005); homolateral median nerve PPTs (F = 0.741; *p* = 0.511; η^2^_p_ = 0.011); contralateral median nerve PPTs (F = 0.140; *p* = 0.890; η^2^_p_ = 0.002)]. All outcomes for median nerve mechanosensitivity are presented in Table 2.

### 3.2. Widespread Pain Assessment

There were significant differences for time factor in the PPT for the homolateral C6 zygapophyseal joint PPT (F = 4.707; *p* = 0.006; η^2^_p_ = 0.065). However, no differences were found in the group × time interaction for pain intensity, body pain distribution, and PPT. Post hoc analysis for pain intensity, body pain distribution, and PPT are shown in Table 3.

### 3.3. Cervical Function and Kinesiophobia

The ANCOVA analysis only found statistically significant differences in the time factor for kinesiophobia (F = 8.013; *p* = 0.001; ŋ^2^_p_ = 0.105). However, no statistically significant differences were observed in the group × time interaction for the cervical function and kinesiophobia. Multiple comparisons for the active cervical ROM, disability, and kinesiophobia are presented in Table 4 and Table 5.

## 4. Discussion

Neural mobilization approaches have been demonstrated to be effective for improving cervicobrachial pain conditions [17,18,19,20]. However, to our knowledge, this is the first study to assess the effectiveness of the combination of two neural mobilization techniques for improving neural mechanosensitivity, pain, and function in patients with NP. Here we demonstrate a positive effect for improving median nerve mechanosensitivity, regardless of whether the application of lateral cervical glide is applied in combination with the mobilization of the median nerve. There are no differences between the two forms. These findings suggest that the application of only one neural mobilization technique might be sufficient to improve NP in clinical practice. Hence, the time spent on manual therapy techniques could be reduced in favor of other alternative approaches, such as therapeutic exercise or therapeutic education [60].

### 4.1. Median Nerve Mechanosensitivity

The amount of improvement obtained in this study is similar or slightly higher than those obtained in other studies. Vicenzino et al. [61] found that patients suffering from epicondylalgia, subjected to a single session of lateral cervical sliding (1.5 min) obtained an improvement of 26.03% (0.45 kg/cm^2^). In our study, we detected an improvement of greater magnitude in the lateral cervical glide group with an increase of 34.16% (0.96 kg/cm^2^) at 30 days of follow-up, while in the group in which lateral cervical glide was combined with the mobilization of the median nerve the percentage of improvement was just 15.94% (0.44 kg/cm^2^). Another more recent study [62] applied three sessions of a combined protocol of manual soft-tissue therapy together with neural mobilization exercises to patients with Carpal tunnel syndrome. These authors detected an improvement in the PPT of the median nerve region of 23.6% (0.80 kg/cm^2^) that remains 1 year after follow-up. Bialosky et al. [63], on the other hand, did not achieve an improvement in mechanical hypoalgesia when applying exercises to mobilize the median nerve (during nine sessions) against a placebo group in patients with carpal tunnel syndrome, although the group subjected to neural mobilization obtained a significant improvement (41.26%), and superior to that found in our study (34.16%). These differences might be because the other study had a greater number of sessions. Finally, a study that applied a session of neural mobilization of the median nerve in patients with NP [64] found contradictory results; there were no improvements in mechanical hypoalgesia in the cervical region. Based on these findings, we propose that the effects of neural mobilization are effects that are best in the body area where it is applied. It could be that the differences with that study are that they did one session while we did two. Moreover, in this research, we found improvements in the mechanical hypoalgesia of the median nerve on the contralateral side, producing improvements of 32.2% and 29.65% in the group of CLG + MNNM and CLG only, respectively.

In relation to neural mechanosensitivity evaluated by the neural tension test neural measured by the range of motion of the elbow extension, Coppieters et al. [65] found an improvement of 13.86% (19 degrees) in patients with cervicobrachial neuralgia when a single session of lateral cervical glide was applied, these results are consistent with those reported in our study. These results are in line with our findings, with a slightly greater improvement in the CLG + MNNM group than CLG group (26.62% and 19.11%, respectively). In contrast, another study [66] found an improvement of only 6% after the application of nine sessions of neural mobilization on the median nerve in healthy patients. It is possible that this difference is because they were not patients suffering from pain.

### 4.2. Widespread Pain Assessment

In relation to the NP intensity, although no differences were found between the groups, these presented an improvement of 27.67% (1.2 points) in the CLG + MNNM group and 31.49% (1.3 points) in the CLG group. These results are lower than those obtained by Rodríguez et al. [19] in which they applied neural mobilization of the median nerve to patients with cervicobrachial neuralgia and obtained a 52.76% improvement (3.08 points], which is much more clinically relevant than the improvements obtained in our study. Although the changes obtained reached the minimally detectable threshold (1.15 points) [67]. The number of sessions that applied was much higher (20 sessions) compared to the two sessions that we applied in our study, which might account for the different findings. Another study [20] in which the authors applied 30 sessions of the lateral cervical sliding technique also found effects on pain intensity superior to our study, with an improvement that reached 35.5% (2.16 points). Finally, in another study [22] that compared 30 sessions of neural mobilization of the median nerve against lateral cervical glide in patients with cervicobrachial neuralgia, the authors found an improvement superior to that of our study, reaching 36.06% (2.2 points) in the group subjected to lateral cervical glide and 46.15% (3 points) in the group of neural mobilization of the median nerve. Again, we believe that the number of sessions is the main cause of this difference. In addition, our study used neural gliding in accordance with prior experimental research which showed that neural mobilization generated immediate widespread hypoalgesic effects in more body sites compared to neural stretching [68].

### 4.3. Cervical Function and Kinesiofobia

Although there was an improvement in function, this was inferior to that reported in other studies. The improvement in rotation in the CLG group only reached improvements of 8.57% (4.96 degrees) and 5.57% (3.32 degrees) in the contralateral rotation. These data are contradictory to those found in several previous studies [19,20,22], achieving a 12.1% improvement in the cervical range of motion in patients with cervicobrachial neuralgia after application of median nerve neural mobilization [19], and an 18% (10 degrees) improvement when lateral cervical slippage was applied [20,22]. This difference could be explained by the fact that, in those three previous studies, 30 sessions were applied as opposed to the two sessions we have applied in our study. Nevertheless, the follow-up presented the same duration (30 days). On the other hand, neck disability was improved in both groups, but neither group was superior to the other according to the results obtained in the NDI. This outcome was assessed with NDI. NDI is the preferred tool for the evaluation of disability levels [49,69] in patients suffering from non-specific NP, with [19,20,21,22] or without irradiation symptoms [50,51,70].

Few studies have previously included the kinesiophobia variable. The two studies we could identify in which neural mobilization is applied used kinesiophobia as a confusion variable to test how it interfered with the improvement of pain in healthy subjects [66] or pain modulation in patients with NP [64]. In our study, all patients improved in kinesiophobia although with discrete changes (2.5 points (8.95%) in the CLG + MNNM group and 3.75 points (13.22%) in the CLG group).

### 4.4. Limitations

This study has some limitations. First, the lack of a control group and/or placebo group means that we cannot determine the effects of these non-specific interventions or test whether they are superior to the absence of treatment. Second, only kinesiophobia was measured, so we cannot determine the effects on other psychological factors. The third limitation is that only two treatment sessions were carried out. If more sessions had been applied, we could have found other effects. Another limitation is the short-term (30 days) follow-up of patients; therefore, we cannot know the effects in the medium and long term.

## 5. Conclusions

Adding neural mobilization of the median nerve to the cervical lateral sliding technique is not superior to performing it alone in improving the mechanosensitivity of the median nerve, expanding pain, NP intensity, improving function, and kinesiophobia. Only two treatment sessions and the short follow-up are important issues, therefore justifying further studies to answer the research question with better methodology.

## Figures and Tables

**Table 1:** Baseline participant characteristics. Values are the mean ± standard deviation.

	CLGS + MNNM Group *n* = (72)	CLGS Group *n* = (72)	*p*-Value
Age, years	44.69 ± 13.48	50.72 ± 9.42	0.03 *
Sex M/F (Female%)	15/21 (48.8%)	14/22 (51.2%)	0.81
Duration of pain (months)	73.11 ± 53.52	61.61 ± 42.09	0.31
PPT-C6-H (kg/cm^2^)	2.48 ± 1.14	2.58 ± 1.09	0.71
PPT-C6-CL (kg/cm^2^)	2.53 ± 0.83	2.74 ± 0.98	0.34
PPT-MN-H (kg/cm^2^)	2.82 ± 0.95	2.91 ± 1.16	0.74
PPT-MN-CL (kg/cm^2^)	2.71 ± 1.01	3.03 ± 1.08	0.20
PPT-AT-H (kg/cm^2^)	7.41 ± 3.40	7.19 ± 3.17	0.77
PPT-AT-CL (kg/cm^2^)	6.99 ± 3.34	7.01 ± 3.20	0.97
Neck-PD	8.45 ± 6.79	8.98 ± 11.67	0.81
UL-PD-HL	5.91 ± 8.38	6.24 ± 12.58	0.89
UL-PD-CL	2.24 ± 4.43	2.63 ± 8.07	0.79
Elbow extension	49.50 ± 19.9	44.36 ± 21.07	0.29
NDI (0 to 50)	33.69 ± 16.64	34.83 ± 16.48	0.77
VAS (0 to 100 mm)	42.86 ± 19.13	42.78 ± 21.33	0.98
CROM (grades)			
Flexion/extension	105.77 ± 29.59	102.30 ± 24.55	0.59
Lateral flexion	69.53 ± 22.25	66.97 ± 17.35	0.58
Rotation	115.58 ± 30.85	115.52 ± 20.99	0.99
Psychological measures			
TSK-11 (11 to 44)	27.31 ± 7.73	28.92 ± 7.94	0.38

Abbreviations: CLGS + MNNM group, cervical lateral glide-side + median nerve neural mobilization; CLGS group, cervical lateral glide-side; PPT-C6-HL, pressure pain threshold on C6 homolateral side; PPT-C6-CL, pressure pain threshold on C6 contralateral side; PPT-AT-H, pressure pain threshold on anterior tibial muscle homolateral side; PPT-AT-CL, pressure pain threshold on anterior tibial muscle contralateral side; VAS, visual analogue scale; SD, standard deviation NDI, neck disability index; CROM, cervical range of motion; 11, tampa scale of kinesiophobia; Neck-PD, pain drawing on neck; UL-PD_HL, upper limb pain drawing homolateral; UL-PD-CL, upper limb pain drawing contralateral. *p* < 0.01. * = *p* value < 0.05.

**Table 2:** Multiple comparisons for median nerve mechanosensitivity. Values are the mean ± standard deviation unless otherwise indicated.

	Group	Baseline	Post-Intervention 5 min	Follow-Up 2 Weeks	Follow-Up 4 Weeks	Mean Difference (95% CI) (a) Baseline vs Post (b) Baseline vs 2-Weeks (c) Baseline vs 4-Weeks	Mean Difference (95% CI) (a) Post vs 2-Weeks (b) Post vs 4-Weeks (c) 2-Weeks vs 4-Weeks
**ULNT1** (degrees for elbow extension)							
Homolateral side pain	CLG + MNNM	50.85 ± 20.78	47.09 ± 20.02	40.80 ± 19.72	37.31 ± 18.47	(a) 3.76 (−0.77 to 8.29) (b) 10.05 (1.54 to 18.56) * (c) 13.55 (5.74 to 21.35) **	(a) 6.29 (−1.53 to 14.11) (b) 9.78 (3.10 to 16.47) ** (c) 3.49 (−2.47 to 9.46)
	CLG	43.58 ± 21.31	41.08 ± 20.52	42.12 ± 20.22	35.25 ± 18.94	(a) 2.50 (−2.15 to 7.14) (b) 1.46 (−7.27 to 10.19) (c) 8.34 (0.34 to 16.34) *	(a) −1.04 (−9.05 to 6.98) (b) 5.84 (−1.01 to 12.69) (c) 6.88 (0.76 to 12.99) *
Contralateral side pain	CLG + MNNM	45.96 ± 18.80	47.13 ± 18.28	38.53 ± 15.98	34.57 ± 16.63	(a) −1.17 (−6.92 to 4.58) (b) 7.43 (−1.30 to 16.16) (c) 11.38 (3.62 to 19.15) **	(a) 8.60 (0.64 to 16.56) * (b) 12.56 (5.15 to 19.97) ** (c) 3.96 (−2.45 to 10.36)
	CLG	43.24 ± 19.27	39.20 ± 18.73	38.27 ± 16.38	32.88 ± 17.05	(a) 4.04 (−1.85 to 9.94) (b) 4.97 (−3.98 to 13.92) (c) 10.37 (2.41 to 18.33) **	(a) 0.93 (−7.23 to 9.10) (b) 6.32 (−1.27 to 13.92) (c) 5.39 (−1.17 to 11.96)
**Median Nerve PPT** (kg/cm^2^)							
Homolateral side pain	CLG + MNNM	2.76 ± 1.06	2.82 ± 1.15	2.89 ± 1.23	3.20 ± 1.36	(a) −0.06 (−0.39 to 0.27) (b) −0.13 (−0.61 to 0.35) (c) −0.44 (−0.95 to 0.07)	(a) −0.07 (−0.53 to 0.39) (b) −0.38 (−0.85 to 0.09) (c) −0.31 (−0.77 to 0.15)
	CLG	2.81 ± 1.09	2.98 ± 1.18	3.19 ± 1.26	3.77 ± 1.39	(a) −0.17 (−0.51 to 0.17) (b) −0.38 (−0.87 to 0.12) (c) −0.96 (−1.48 to −0.43) **	(a) −0.21 (−0.68 to 0.27) (b) −0.79 (−1.27 to −0.31) ** (c) −0.58 (−1.05 to −0.11) **
Contralateral side pain	CLG + MNNM	2.67 ± 1.03	2.81 ± 1.04	3.04 ± 1.08	3.53 ± 1.43	(a) −0.15 (−0.47 to 0.18) (b) −0.37 (−0.84 to 0.09) (c) −0.86 (−1.45 to −0.28) **	(a) −0.23 (−0.70 to 0.24) (b) −0.72 (−1.31 to −0.12) * (c) −0.49 (−0.96 to −0.01) *
	CLG	2.90 ± 1.06	3.13 ± 1.07	3.33 ± 1.11	3.76 ± 1.47	(a) −0.23 (−0.56 to 0.10) (b) −0.44 (−0.91 to 0.04) (c) −0.86 (−1.47 to −0.26) **	(a) −0.21 (−0.69 to 0.28) (b) −0.64 (−1.24 to −0.03) * (c) −0.43 (−0.92 to 0.06)

Abbreviations: CLG, cervical lateral glide; MNNM, median nerve neural mobilization; PPT, pressure pain threshold; ULNT1, upper limb neurodynamic test. * = *p* value < 0.05, ** = *p* value < 0.01.

**Table 3:** Multiple comparisons for pain intensity, symptom distribution and pressure pain thresholds. Values are the mean ± standard deviation unless otherwise indicated.

	Group	Baseline	Post-Intervention 5 min	Follow-Up 2 Weeks	Follow-Up 4 Weeks	Mean Difference (95% CI) (a) Baseline vs Post (b) Baseline vs 2-Weeks (c) Baseline vs 4-Weeks	Mean Difference (95% CI) (a) Post vs 2-Weeks (b) Post vs 4-Weeks (c) 2-Weeks vs 4-Weeks
VAS (0–100 mm)	CLG + MNNM	44.19 ± 20.61	40.71 ± 21.88	42.29 ± 21.64	31.96 ± 19.89	(a) 3.48 (−4.41 to 11.38) (b) 1.90 (−6.77 to 10.56) (c) 12.23 (3.01 to 21.44) **	(a) −1.59 (−10.42 to 7.25) (b) 8.74(−0.84 to 18.33) (c) 10.33 (3.30 to 17.36) **
	CLG	42.39 ± 21.13	38.08 ± 22.43	36.60 ± 22.19	29.04 ± 20.39	(a) 4.30 (−3.79 to 12.40) (b) 5.79 (−3.09 to 14.67) (c) 13.35 (3.89 to 22.80) **	(a) 1.48 (−7.58 to 10.55) (b) 9.04 (−0.78 to 18.87) (c) 7.56 (0.35 to 14.76) *
**Body pain distribution** (pain expansion drawing)							
Neck pain	CLG + MNNM	8.27 ± 9.92	5.64 ± 7.06	7.89 ± 8.45	3.93 ± 3.95	(a) 2.63 (−0.84 to 6.10) (b) 0.38 (−3.79 to 4.56) (c) 4.34 (0.12 to 8.57) *	(a) −2.25 (−6.04 to 1.55) (b) 1.72 (−1.23 to 4.66) (c) 3.96 (0.61 to 7.32) *
	CLG	8.92 ± 10.17	6.36 ± 7.24	6.71 ± 8.66	4.43 ± 4.05	(a) 2.57 (−0.99 to 6.12) (b) 2.21 (−2.07 to 6.49) (c) 4.49 (0.16 to 8.83) *	(a) −0.36 (−4.24 to 3.53) (b) 1.93 (−1.09 to 4.94) (c) 2.28 (−1.16 to 5.72)
**PPT** (kg/cm^2^)							
Homolateral C6	CLG + MNNM	2.41 ± 1.12	2.46 ± 1.07	2.70 ± 1.11	3.25 ± 1.22	(a) −0.06 (−0.41 to 0.30) (b) −0.30 (−0.81 to 0.22) (c) −0.84 (−1.38 to −0.30) **	(a) −0.24 (−0.71 to 0.23) (b) −0.79 (−1.25 to −0.32) ** (c) −0.55 (−1.00 to −0.09) *
	CLG	2.51 ± 1.15	2.81 ± 1.10	3.06 ± 1.13	3.66 ± 1.25	(a) −0.30 (−0.67 to 0.07) (b) −0.56 (−1.09 to −0.02) * (c) −1.15 (−1.71 to −0.60) **	(a) −0.25 (−0.74 to 0.23) (b) −0.85(−1.33 to −0.37) ** (c) −0.60 (−1.07 to −0.13) **
Contralateral C6	CLG + MNNM	2.47 ± 0.91	2.63 ± 1.15	2.80 ± 0.97	3.40 ± 1.27	(a) −0.15 (−0.48 to 0.18) (b) −0.33 (−0.76 to 0.10) (c) −0.93 (−1.44 to −0.42) **	(a) −0.18 (−0.59 to 0.23) (b) −0.78(−1.22 to −0.33) ** (c) −0.60 (−0.98 to −0.22) **
	CLG	2.68 ± 0.94	2.86 ± 1.18	3.08 ± 1.00	3.65 ± 1.30	(a) −0.18 (−0.52 to 0.16) (b) −0.40 (−0.84 to 0.04) (c) −0.98 (−1.50 to −0.46) **	(a) −0.22 (−0.64 to 0.20) (b) −0.80 (−1.25 to −0.34) ** (c) −0.58 (−0.97 to −0.18) **
Tibial muscle	CLG + MNNM	6.91 ± 3.06	6.92 ± 3.16	7.77 ± 2.96	7.95 ± 2.78	(a) −0.01 (−0.56 to 0.55) (b) −0.86 (−1.75 to 0.03) (c) −1.04 (−1.93 to −0.15) *	(a) −0.85 (−1.78 to 0.08) (b) −1.04(−1.88 to −0.19) ** (c) −0.18 (−0.98 to 0.61)
	CLG	7.24 ± 3.14	7.88 ± 3.24	7.59 ± 3.04	7.80 ± 2.85	(a) −0.64 (−1.20 to −0.08) * (b) −0.35 (−1.26 to 0.56) (c) −0.55 (−1.46 to 0.36)	(a) 0.29 (−0.66 to 1.24) (b) 0.09 (−0.78 to 0.95) (c) −0.20 (−1.02 to 0.61)

Abbreviations: CLG, cervical lateral glide; MNNM, median nerve neural mobilization; PPT, pressure pain threshold; VAS, visual analogue scale; C6-zygapophyseal joint, sixth zygapophyseal cervical joint, * = *p* value < 0.05, ** = *p* value < 0.01.

**Table 4:** Multiple comparisons for active cervical range of motion. Values are the mean ± standard deviation unless otherwise indicated.

CROM (Degrees)	Group	Baseline	Post-Intervention 5 min	Follow-Up 2 Weeks	Follow-Up 4 Weeks	Mean Difference (95% CI) (a) Baseline vs Post (b) Baseline vs 2-Weeks (c) Baseline vs 4-Weeks	Mean Difference (95% CI) (a) Post vs 2-Weeks (b) Post vs 4-Weeks (c) 2-Weeks vs 4-Weeks
Flexion/ Extension	CLG + MNNM	101.51 ± 23.53	100.86 ± 23.76	100.21 ± 20.35	101.71 ± 17.74	(a) 0.65 (−5.19 to 6.50) (b) 1.30 (−6.58 to 9.18) (c) −0.20 (−8.33 to 7.93)	(a) 0.65 (−6.48 to 7.77) (b) −0.85 (−8.04 to 6.33) (c) −1.50 (−7.95 to 4.95)
	CLG	105.37 ± 24.12	109.14 ± 24.35	108.76 ± 20.86	107.65 ± 18.18	(a) −3.77 (−9.76 to 2.22) (b) −3.39 (−11.47 to 4.69) (c) −2.28 (−10.61 to 6.05)	(a) 0.38 (−6.93 to 7.68) (b) 1.49 (−5.88 to 8.85) (c) 1.11 (−5.51 to 7.72)
Homolateral Rotation	CLG + MNNM	55.26 ± 13.08	58.23 ± 13.40	54.82 ± 11.49	55.94 ± 12.07	(a) −2.98 (−7.43 to 1.48) (b) 0.44 (−4.52 to 5.39) (c) −0.68 (−5.16 to 3.80)	(a) 0.41 (−1.53 to 8.35) (b) 2.30 (−2.93 to 7.52) (c) −1.12 (−5.13 to 2.90)
	CLG	57.87 ± 13.41	60.11 ± 13.74	59.53 ± 11.78	62.83 ± 12.38 *	(a) −2.24 (−6.81 to 2.33) (b) −1.66 (−6.74 to 3.43) (c) −4.95 (−9.55 to −0.36) *	(a) 0.58 (−4.49 to 5.65) (b) −2.72 (−8.07 to 2.64) (c) −3.30 (−7.41 to 0.82)
Contralateral Rotation	CLG + MNNM	56.17 ± 12.05	60.18 ± 12.71	57.45 ± 11.70	55.07 ± 12.38	(a) −4.01 (−8.64 to 0.62) (b) −1.27 (−6.08 to 3.53) (c) 1.10 (−3.42 to 5.61)	(a) 2.74 (−2.19 to 7.66) (b) 5.11 (0.39 to 9.82) * (c) 2.37 (−1.28 to 6.02)
	CLG	59.51 ± 12.35	61.22 ± 13.03	61.30 ± 12.00	62.83 ± 12.70 *	(a) −1.71 (−6.46 to 3.04) (b) −1.80 (−6.72 to 3.13) (c) −3.32 (−7.95 to 1.31)	(a) −0.09 (−5.13 to 4.96) (b) −1.61 (−6.44 to 3.23) (c) −1.52 (−5.27 to 2.22)
Homlateral side flexion	CLG + MNNM	33.81 ± 9.42	35.65 ± 11.20	34.03 ± 9.65	35.12 ± 8.77	(a) −1.84 (−5.19 to 1.51) (b) −0.22 (−3.54 to 3.10) (c) −1.31 (−4.71 to 2.09)	(a) 1.62 (−2.06 to 5.31) (b) 0.53 (−3.35 to 4.41) (c) −1.09 (−4.06 to 1.88)
	CLG	33.85 ± 9.66	36.93 ± 11.48	34.35 ± 9.89	35.69 ± 8.99	(a) −3.08 (−6.51 to 0.35) (b) −0.50 (−3.90 to 2.90) (c) −1.84 (−5.32 to 1.65)	(a) 2.58 (−1.19 to 6.36) (b) 1.24 (−2.73 to 5.22) (c) −1.34 (−4.38 to 1.71)
Contralateral side flexion	CLG + MNNM	33.12 ± 10.93	34.45 ± 10.86	34.60 ± 10.27	36.52 ± 9.86	(a) −1.33 (−4.91 to 2.25) (b) −1.48 (−6.03 to 3.06) (c) −3.40 (−7.56 to 0.76)	(a) −0.15 (−3.69 to 3.39) (b) −2.07 (−5.57 to 1.44) (c) −1.92 (−5.67 to 1.84)
	CLG	34.61 ± 11.21	35.36 ± 11.14	35.93 ± 10.53	37.00 ± 10.10	(a) −0.76 (−4.43 to 2.92) (b) −1.32 (−5.98 to 3.34) (c) −2.39 (−6.65 to 1.88)	(a) −0.57 (−4.19 to 3.06) (b) −1.63 (−5.23 to 1.96) (c) −1.07 (−4.92 to 2.78)

Abbreviations: CLG, cervical lateral glide; MNNM, median nerve neural mobilization; CROM, cervical range of motion. * = *p* value < 0.05.

**Table 5:** Multiple comparisons for neck disability and kinesiophobia. Values are the mean ± standard deviation unless otherwise indicated.

	Group	Baseline	Follow-Up 2 Weeks	Follow-Up 4 Weeks	Mean Difference (95% CI) (a) Baseline vs 2-weeks (b) Baseline vs 4-weeks (c) 2-weeks vs 4-weeks
NDI	CLG + MNNM	17.66 ± 7.94	15.69 ± 7.31	13.88 ± 6.98	(a) 1.97 (0.28 to 3.66) * (b) 3.79 (1.63 to 5.95) ** (c) 1.82 (0.38 to 3.26) **
	CLG	16.54 ± 8.14	14.16 ± 7.49	11.73 ± 7.16	(a) 2.38 (0.65 to 4.11) ** (b) 4.81 (2.60 to 7.03) ** (c) 2.44 (0.96 to 3.91) **
TSK-11	CLG + MNNM	27.93 ± 7.78	25.21 ± 7.78	25.43 ± 7.70	(a) 2.73 (0.80 to 4.65) ** (b) 2.50 (0.33 to 4.67) * (c) −0.23 (−2.14 to 1.69)
	CLG	28.35 ± 7.98	26.81 ± 7.98	24.60 ± 7.90	(a) 1.54 (−0.44 to 3.51) (b) 3.75 (1.52 to 5.97) ** (c) 2.21 (0.25 to 4.18) *

Abbreviations: CLG, cervical lateral glide; NDI, neck disability index; MNNM, median nerve neural mobilization; TSK−11, Tampa Scale of Kinesiophobia. * = *p* value < 0.05, ** = *p* value < 0.01.

## Data Availability

Data available on request due to privacy and ethical restrictions.
